# Single-level anterior cervical discectomy and interbody fusion using PEEK anatomical cervical cage and allograft bone

**DOI:** 10.1007/s10195-011-0169-4

**Published:** 2011-11-17

**Authors:** C. Faldini, M. Chehrassan, M. T. Miscione, F. Acri, M. d’Amato, C. Pungetti, D. Luciani, S. Giannini

**Affiliations:** Department of Orthopaedic Surgery, Istituto Ortopedico Rizzoli, University of Bologna, Via G.C. Pupilli 1, 40136 Bologna, Italy

**Keywords:** ACDF, PEEK cage, Allograft bone

## Abstract

**Background:**

In an effort to avoid the morbidity associated with autogenous bone graft harvesting, cervical cages in combination with allograft bone are used to achieve fusion. The goal of the current study was to assess the reliability and efficacy of anterior cervical discectomy and interbody fusion (ACDF) using a PEEK anatomical cervical cage in the treatment of patients affected by single-level cervical degenerative disease.

**Methods and materials:**

Twenty-five patients affected by single-level cervical degenerative pathology between C4 and C7 were enrolled in this study. The clinical findings were assessed using the Neck Disability Index and the Visual Analog Scale. Surgical outcomes were rated according to Odom’s criteria at last follow-up. Fusion was graded as poor, average, good or excellent by assessing the radiographs. Cervical spine alignment was evaluated by sagittal segmental alignment and sagittal alignment of the whole cervical spine preoperatively, 6 months postoperatively and at the last follow-up.

**Results:**

Twenty-five patients underwent ACDF using a PEEK anatomical cervical cage. All patients had a minimum 2 years of follow-up. The operative levels were C4–C5 in 5 patients, C5–C6 in 12 patients and C6–C7 in 8 patients. Preoperatively, average NDI was 34, 13 at 6 months, and 10 at latest follow-up. The mean preoperative VAS was 7; the mean postoperative VAS at latest follow-up was 3. Good or excellent fusion was achieved in all patients within 10 months (mean 5 months). Preoperatively, average sagittal segmental alignment (SSA) was 0.2° and average sagittal alignment of the cervical spine (SACS) 15.8°. Six months after surgery, average SSA was 1.8° and average SACS 20.9°, and at last follow-up, average SSA was 1.6° and average SACS 18.5°.

**Conclusion:**

Anterior cervical discectomy and interbody fusion using PEEK anatomical cervical cages can be considered a safe and effective technique to cure cervical disc herniation with intractable pain or neural deficit in cases where conservative treatment failed.

## Introduction

Degenerative disease of the cervical spine is a common cause of neck and upper limb pain which, in severe cases, could potentially be a debilitating disease. In addition to age-related degenerative changes, there are many other conditions that could lead to degenerative changes at the level of the cervical spine [8, 18, 36]. Several procedures have been described for the treatment of disc herniation and cervical spondylosis when conservative treatment fails, including anterior decompression, laminectomy, laminoplasty and instrumented anterior and posterior fusion by plates or screws [2, 3, 5, 6, 10, 13, 14, 21, 22, 24, 39]. Anterior cervical discectomy and interbody fusion (ACDF) is a surgical technique used to treat a variety of cervical spine disorders, such as nerve root or spinal cord compression, cervical spondylosis, and cervical spinal stenosis [9, 15]. The anterior approach to the cervical spine for discectomy and fusion by the insertion of an autologous iliac-crest tricortical bone graft was first described by Robinson and Smith in 1955 [39]. In 1958, Cloward described a wide anterior cylindrical discectomy performed with a special reamer combined with anterior fusion by the insertion of autologous iliac bone graft of the same shape [13]. Several implants used to perform anterior interbody fusion were later described. Bagby et al. developed the first interbody fusion cage [2]. Cages of different shapes and materials are used to perform ACDF which, in some cases, could be associated with plate fixation [8, 11, 12, 17, 20].

The aim of the current study was to determine whether an anterior cervical discectomy and fusion with a polyetheretherketone (PEEK) anatomical cervical cage filled with allograft bone to perform fusion could be effective for decompressing the spinal cord, recovering cervical sagittal alignment, and providing solid arthrodesis and relief from symptoms with minimal surgical risk.

## Materials and methods

A retrospective clinical study of 25 patients affected by single-level degenerative pathology between C4 and C7 who had undergone ACDF, between 2007 and 2009 was performed. The inclusion criterion for this study was single-level degenerative cervical pathology. In the case of spondylosis, the diagnosis was made based on the cervical degenerative index [35]. Indication for surgery was cervical pain associated with intractable radioculopathy that did not respond to nonoperative (conservative) treatment for a period of at least six weeks, or demonstrating progressive neurologic deficit during a period of observation. Patients with fractures, infection, deformity, tumors, chronic systemic illnesses such as diabetes mellitus, rheumatoid arthritis, and neurodegenerative diseases, or those with disorders at more than one level were excluded from this study. No exclusion was made based on sex, age, or the intensity of the preoperative clinical signs. Before surgery, all patients had plain AP and lateral radiographs, a CT scan or an MRI scan of their cervical spine.

Surgery consisted of single-level anterior discectomy and interbody fusion. To perform the intervention, the patient was placed on a surgical bed in the supine position with the neck extended slightly; under general anesthesia, an anterior–oblique longitudinal approach was used, overlying the medial border of the sternocleidomastoid muscle at the level of the degenerated intervertebral disc. The trachea and esophagus were retracted medially and the neurovascular bundle with the sternocleidomastoid muscle laterally. After fluoroscopic confirmation of the affected level, a complete discectomy and decompression was performed. The cervical column was placed in physiologic lordosis with the help of a Caspar screw distractor; then a PEEK anatomical cervical cage was inserted into the intervertebral space. Before the insertion, the cage was filled with cancellous bone allograft chips which were provided by Bone Bank. The postoperative protocol included discharge 1 day after surgery with soft collar protection for 3 weeks. The rehabilitation program included transcutaneous electrical nerve stimulation therapy (TENS) and local gentle massage during this period. After 3 weeks, the patient was checked in the outpatient facility, the soft collar was removed, and light recovery of the cervical spine with a physiotherapist was advised [8, 12, 13, 15, 17, 18, 22, 29].

**Fig. 1:**
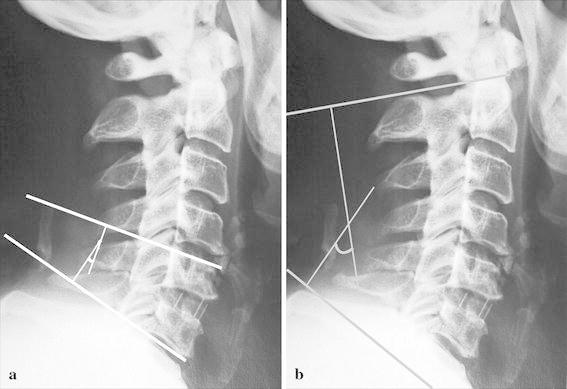
**a** Postoperative sagittal segmental alignment (SSA) angle, and **b** postoperative sagittal alignment of the cervical spine (SACS) angle

The clinical findings were assessed using the Neck Disability Index (NDI) [38, 46] and the Visual Analog Scale (VAS) [41] both pre- and postoperatively. At the last follow-up, the outcomes were rated according to Odom’s criteria as excellent, good, fair, or poor, depending on the resolution, improvement, or persistence of preoperative symptoms (Table 1) [34]. At 6 months and at the last follow-up, the fusion was graded by assessing the radiographs as poor, average, good, or excellent. Fusion was confirmed by the presence of continuous trabecular bone bridges in at least one of the following locations: anterior, within, or posterior to the cage. The absence of such bridges or the presence of an anterior–posterior discontinuation was classified as nonfusion [37]. Cervical spine alignment was evaluated by sagittal segmental alignment (SSA) and sagittal alignment of the cervical spine (SACS) on lateral radiographs, preoperatively, 6 months postoperatively, and at the last follow-up (Fig. 1) [1, 15, 16].

**Table 1:** Classification of outcome according to Odom’s criteria

Excellent	All preoperative symptoms relieved; abnormal findings improved
Good	Minimal persistence of preoperative symptoms; abnormal findings unchanged or improved
Fair	Definite relief of some preoperative symptoms; other symptoms unchanged or slightly improved
Poor	Symptoms and signs unchanged or exacerbated

All patients had a minimum 2 years of follow-up.

The study conforms to the Declaration of Helsinki as revised in 2008 and was approved by the institutional committee for medical ethics. All patients provided informed written consent.

## Results

A total of 25 patients (3 female, 22 male) with a mean age of 42 (aged between 33 and 60 years) underwent ACDF through the use of PEEK anatomical cages filled with allograft bone. The operative levels were C4–C5 in 5 patients (20%), C5–C6 in 12 patients (48%), and C6–C7 in 8 patients (32%). Twenty-two patients (88%) presented with radiculopathy due to cervical disc herniation and 3 patients (12%) due to cervical spondylosis. Twenty patients were discharged the day after surgery; 5 patients remained in the hospital for more than 2 days, including 3 for sustained postoperative pain and 2 with suspected postsurgical hematoma. All 5 of these patients were discharged without any complications 3 days after surgery.

The average NDI preoperatively was 34 (range 31–50), at 6 months it was 13 (range 3–22), and at the latest follow-up it was 10 (range 0–22). The mean preoperative VAS was 7 (range 4–10); the mean postoperative VAS at latest follow-up was 3 (range 0–5). The Neck Disability Index and Visual Analog Scale scores had both improved significantly in all patients by the last follow-up.

Odom’s criteria for grading relief from symptoms was applied at the last follow-up. Nine patients (36%) presented with an excellent clinical outcome, 11 good (44%), 5 fair (20%), and no patient presented a poor outcome. Twenty patients (80%) showed clear relief from preoperative symptoms with subsequent functional improvement.

The mean time taken to achieve a grading of at least good radiographic signs of fusion in our study was 5 months (range 3–10). Good or excellent fusion was achieved in all patients within 10 months.

Preoperatively, the average SSA was 0.2° ± 2.2, and the average SACS 15.8° ± 3.8. As seen in the radiographs taken 6 months postoperatively, the average SSA was 1.8° ± 3.8 and the average SACS 20.9° ± 5.8. At last follow-up, the average SSA was 1.6° ± 4.6 and the average SACS 18.5° ± 6.0.

All patients had healed uneventfully with good results by the 2-year follow-up. No serious complications—including deaths, reoperation, neurological damage (permanent or temporary), Horner’s syndrome, pseudarthrosis, hardware failure, infection, and thrombosis—were seen.

All patients were permitted to return to light work by 4 weeks, and were allowed to perform heavier work and sports within 2–3 months after surgery.

There were no statistically significant differences in sex and age distribution.

## Discussion

Anterior cervical discectomy and decompression with interbody fusion can be a good surgical choice when conservative treatment for cervical disc herniation or cervical spondylosis fails [7, 13, 15, 40]. Although tricortical autograft harvested from the iliac crest as interbody fusion material can provide satisfactory clinical results and fusion rates [15, 25], complication rates at the donor site are around 20% [42, 43], and could be a potential disadvantage of this technique. Interbody cages provide initial stability and, by filling the disc space, require less structural bone graft and consequently reduce the morbidity associated with autogenous bone graft harvesting [2, 23, 27, 42]. Different types of cages are available to perform ACDF, including titanium cages, carbon fiber reinforced polymer (CFRP) cages, and polyetheretherketone (PEEK) cages. Donor site complications can be omitted by making use of all of these cage types. Titanium cages can provide mechanical support, initial disc height maintenance, and restoration of sagittal lordosis; however, unfavorable outcomes were reported in some studies [11, 20, 33]. Kolstad et al. [27] reported several unfavorable outcomes following radiographic parameter analysis after ACDF using a cylindrical titanium cage. In another study, subsidence or migration of the titanium cage were observed, resulting in disc height collapse and kyphotic deformity [32]. Furthermore, metallic cages are radioopaque, which prevents clear observation of trabecular bone formation and of radiographic fusion signs. Carbon fiber cages (CFC) can be safe and effective, and can lead to restoration of segmental alignment and solid fusion [31, 45]. However, high rates of subsidence have been reported following ACDF using CFC (29.2%) in some studies [4].

The absence of cytotoxicity and mutagenicity were demonstrated for a polyetheretherketone (PEEK) cage in an in vitro study [26]. With biocompatible, nonabsorbable, and corrosion-resistant abilities, the PEEK cage is thought to be a safe biomaterial spacer for spine surgery [44]. Moreover, the modulus of elasticity of PEEK is similar to that of bone [47]. This distinguishing feature is thought to be able to prevent cage subsidence induced by metallic cages. In an in vitro biomechanical study, the stiffness of the PEEK cage was statistically higher than that of the normal motion segment in flexion. The volume-related stiffness of the PEEK cage was higher than that of iliac bone in all directions. These results show that polyetheretherketone could be manufactured as the optimal interbody spacer, providing an adequate volume for bone refilling and immediate mechanical stability in ACDF [19, 30]. The PEEK cage is radiolucent and allowing the surgeon to better evaluate fusion status on radiographs or CT scans. In our series, all 25 patients (100%) achieved good solid fusion within 10 months (mean 5 months) using a PEEK cage filled with cancellous allograft bone chips. These results confirm those of other studies [28, 30]. In addition to a high fusion rate, successful treatment depends on the maintenance of interspace height and segmental angle [16, 25, 30]. Sagittal segmental alignment and sagittal alignment of the whole cervical spine are good indicators of the efficacy of anterior cervical discectomy and interbody fusion [16]. Comparison between preoperative and postoperative SSA and SACS demonstrated the efficacy of our technique for correcting cervical sagittal alignment when degenerative changes produce cervical spine straightening or cervical kyphosis. SSA and SACS angles measured at last follow-up demonstrate a slight loss of correction in comparison with the 6 month postoperative angles, but these changes were not significant, suggesting that the correction obtained with surgery was maintained even after 2 years.

Good or excellent results according to Odom’s criteria in 80% of patients and significant improvements in the VAS and NDI assessments at last follow-up demonstrate that ACDF can be considered a good and effective technique for treating patients suffering from degenerative cervical pathology, and can improve the quality of life at short- and long-term follow-up.

The restoration of segmental alignment in lordosis with high rates of fusion in association with minimal surgical risk in this group of patients, while avoiding the complication of donor site bone graft harvesting, encourages the authors to use this technique more widely.

In conclusion, in order to reduce pain in and improve the quality of life of patients with degenerative cervical disc disease, disc herniation or cervical spondylosis where conservative treatment has failed, anterior cervical discectomy and interbody fusion using PEEK anatomical cervical cages can be an effective and low-risk technique. Filling the cages with allograft bone provides a good grade of fusion and solid arthrodesis. Our technique for ACDF with a PEEK anatomical cervical cage allows decompression of the spinal cord and nerve roots in combination with interbody fusion to provide segmental alignment in lordosis and solid arthrodesis with minimal surgical risk.
